# Iodide Excess Inhibits Thyroid Hormone Synthesis Pathway Involving XBP1-Mediated Regulation

**DOI:** 10.3390/nu15040887

**Published:** 2023-02-09

**Authors:** Jing Yu, Siyi Shen, Ying Yan, Lingxiao Liu, Rongkui Luo, Shengnan Liu, Yuting Wu, Yuying Li, Jingjing Jiang, Hao Ying

**Affiliations:** 1 CAS Key Laboratory of Nutrition, Metabolism and Food Safety, Shanghai Institute of Nutrition and Health, University of Chinese Academy of Sciences, Chinese Academy of Sciences, and Shanghai Jiao Tong University Affiliated Sixth People’s Hospital, Shanghai 200233, China; 2 Innovation Center for Intervention of Chronic Disease and Promotion of Health, Shanghai 200025, China; 3 Department of Interventional Radiology, National Clinical Research Center for Interventional Medicine, Zhongshan Hospital, Fudan University, Shanghai 200032, China; 4 Department of Pathology, Zhongshan Hospital, Fudan University, Shanghai 200032, China; 5 Department of Endocrinology and Metabolism, Zhongshan Hospital, Fudan University, Shanghai 200032, China; 6 Key Laboratory of Food Safety Risk Assessment, Ministry of Health, Beijing 100021, China

**Keywords:** iodide excess, thyroid organoids, thyroid hormone synthesis, ER stress

## Abstract

Iodine is an essential micronutrient for producing thyroid hormone (TH); however, iodide excess can lead to adverse thyroidal effects. Unfortunately, the lack of a proper in vitro model system hampered the studies of the effect of iodide excess on thyroid physiology and pathology. Here, we demonstrated that excessive iodide intake downregulated the genes related to TH synthesis in the thyroids of mice. Since sodium iodide has no effect on these genes in cultured cell lines, we developed a three-dimensional (3D) culture system to enable the murine thyrocytes to form organoids in vitro with thyroid follicle-like structures and function and found that the in vivo effect of iodide excess could be mimicked in these thyroid organoids. Our data indicate that iodide excess mainly activated the XBP1-mediated unfolded protein response in both murine thyroid and thyroid organoids, while activation of XBP1 was able to mimic the sodium iodide effect on genes for the synthesis of TH in murine thyroid organoids. Lastly, our results suggest that XBP1 might transcriptionally repress the genes involved in the synthesis of TH. Based on these findings, we propose that iodide excess inhibits the transcription of genes related to TH synthesis through a mechanism involving XBP1-mediated action.

## 1. Introduction

The main function of the thyroid gland is to synthesize and secrete thyroid hormone (TH), which is important for growth, differentiation, development, and metabolism [1]. Iodine is an intrinsic component of TH [2], and iodide is the only iodine-derived anion species that can be transported by sodium/iodide symporter (NIS) on the membrane of follicular cells of the thyroid. The follicular cells sequester iodide and synthesize thyroglobulin (Tg), which is secreted into the lumen of thyroid follicles. The release of TH is achieved after the luminal Tg undergoes reuptake, proteolysis, and lysosomal degradation. Diet is a main source of iodine, including iodized salt, dairy products (owing to the application of iodophor cleaners), some bread (owing to the iodate bread conditioners application), and seafood (e.g., seaweed) [3]. Although the thyroid gland has the capacity to maintain normal TH homeostasis [1], epidemiological studies have uncovered that excessive iodine intake can give rise to thyroid dysfunction, including hypo- and hyperthyroidism, goiter, and thyroid autoimmunity (Hashimoto’s disease) [4]. It has long been believed that the thyroid gland transiently reduces hormone production to alleviate the toxicity of excessive iodide, which is referred to as the acute Wolff–Chaikoff effect [3]. Although the escape from or the adaptation to acute the Wolff–Chaikoff effect can be partially explained by markedly decreased NIS, the underlying mechanisms are not completely understood [3,5,6].

Endoplasmic reticulum (ER) is a dynamic intracellular organelle, which is crucial for cellular homeostasis and stress response. One of the major functions of ER is to serve as a quality control system and maintain protein homeostasis by coordinating the folding, sorting, trafficking, and degrading pathways for newly synthesized proteins [7,8,9]. Many kinds of disturbances can cause the dysfunction of the ER, leading to a large amount of unfolded or misfolded proteins accumulated in the lumen, which is regarded as ER stress [10]. In response to ER stress, cells activate unfolded protein response (UPR) as an adaptive mechanism to withstand the ER stress. ER stress is a characteristic of highly specialized secretory cells, in which both protein synthesis and secretion are in high demand. Accordingly, the role of key components (inositol-requiring enzyme 1α (IRE1α), X-box-binding protein 1 (XBP1), etc.) in UPR has been implicated in regulating cellular homeostasis in secretory cells [11].

A large number of secretory proteins are produced during the TH synthesis; therefore, thyroid follicles are highly sensitive to factors causing ER stress [12,13]. Recent studies showed that inducing ER stress by tunicamycin in cultured rat thyrocytes (FRTL-5 cells) can downregulate multiple genes related to TH synthesis, such as thyroid stimulating hormone receptor (TSHR), sodium/iodide symporter (NIS), thyroid peroxidase (TPO), and thyroglobulin (Tg) [14,15]. However, probably due to lacking a proper culture system for in vitro modeling, whether and how iodide excess has an impact on ER stress in thyrocytes and thereby affects the function of thyroid follicles are largely unknown. Here, by taking advantage of thyroid organoids, we demonstrate that iodide excess inhibits the mRNA expression of genes for TH synthesis via a mechanism involving XBP1-mediated regulation.

## 2. Materials and Methods

### 2.1. Animals and Treatments

The TPO-CreER^T2^ and ROSA26-tdTomato mice were both from Jackson Laboratory (no. 026512 and no. 007909). The iodide excess model was established by using adult male C57BL/6J mice and the addition of sodium iodide in their drinking water (500 μg/g) for 3 months. Then the mice receiving a high-iodide diet (HID group) were compared to the control group (CT group) (*n* = 9 mice in each group). Pooled samples, each containing thyroids pooled from 3 mice in each group, were used for analysis. The Rosa26^tdTomato/+^; TPO^CreERT2/+^ mice and Rosa26^tdTomato/−^;TPO^CreERT2/+^ mice were intraperitoneally injected with tamoxifen (100mg/kg) (Sigma, T5648-5G). Mice were housed in a specific pathogen-free animal facility under standard conditions. The sequences of the genotyping primers specific to TPO-CreER^T2^ and ROSA26-tdTomato, as well as the band sizes of PCR products, are provided (Table 1).

### 2.2. Culture and Treatment of Cells and Organoids

Nthy-ori 3-1 cells (ATCC) were cultured in RPMI 1640 media containing 10% fetal bovine serum and 1% GlutaMAX. BCPAP cells (ATCC) were cultured in RPMI 1640 media containing 10% fetal bovine serum, 1% GlutaMAX, 1% non-essential amino acids, and 1% sodium pyruvate. HEK293T cells (ATCC) were cultured in DMEM media supplemented with 10% fetal bovine serum. To explore the effect of iodide excess, cultured monolayer cells were treated with PBS, 10 nM NaI, 1 µM NaI, 100 µM NaI, or 10 mM NaI for 72 h before collected for qRT-PCR, as indicated.

The isolation and culture of thyroid organoid from mice thyroid glands were performed as previously described [16,17,18]. Briefly, fresh thyroid tissues of 6–8-week-old mice were sequentially digested at 37 °C with Collagenase type II (Life Technologies, New York, NY, USA, 17101-015, 5 mg/mL) and Y-27632 dihydrochloride (MCE, Monmouth Junction, NJ, USA, HY-10583, 10 µM) for an hour and with TrypLE (Gibco, New York, NY, USA, 12605-010) and Y-27632 (MCE, HY-10583, 10 µM) for 15 min.

For organoid culture, primary mouse thyroid cells were suspended by culture medium mixed with Matrigel at a ratio of 1:1 and seeded into a prewarmed 24-well culture plate. The culture medium consisted of Advanced DMEM/F-12 (Gibco, 12634010) supplemented with 1% HEPES (Life Technologies, 15630-056), 1% GlutaMAX (Life Technologies, 35050-068), 1× B27 supplement (Life Technologies, 17504-044), 1.25 mM *N*-acetyl-L-cysteine (Sigma-Aldrich, Darmstadt, Germany, A9165), 500 ng/mL recombinant human R-spondin-1 (Peprotech, Cranbury, NJ, USA, 120-38), 100 ng/mL recombinant human Noggin (Peprotech, 120-10C), 50 ng/mL human EGF (Peprotech, AF-100-15), 100 ng/mL human FGF10 (Peprotech, 100-26), 200 nM A83-01 (Tocris, Minneapolis, MN, USA, 2939), and 1 mU/mL hTSH (Sigma, Darmstadt, Germany, T9265). In addition, 10 μM Y-27632 (MCE, HY-10583) was added for the first 7 days of culture. For organoid passaging, organoids were collected and released from the Matrigel, using Collagenase Dispase (Roche, Basel, Switzerland, 10269638001) at 37 °C, with shaking, for 30 min; then they were dissociated into single cells with 1 mL TrypLE at 37 °C, with shaking, for 15 min. For tdTomato expression analysis, organoids were treated with 1 μM tamoxifen for 24 h after culturing for 14 days.

To assess the function of thyroid organoids, organoids were incubated for 5 days with refreshed medium containing 100 nM NaI with or without hTSH to measure free T4 in the culture supernatants, using mouse fT4 ELISA kit (San Diego, CA, USA, MBS765283). Dual-Luciferase^@^ Reporter Assay System (Promega, E1910) was used for luciferase assay [19]. HEK293T cells co-transfected with pcDNA3.1 vector or TH receptor β (TRβ) and Pal-luc plasmids (a luc-reporter containing palindromic TH response elements) were treated with Td medium or the culture supernatants (CS) of organoids for 12–16 h. Cells were then lysed, and luciferase activity measured.

To evaluate the iodide effects, thyroid organoids were incubated with culture media containing PBS, 100 µM NaI or 10 mM NaI for 24 h. Organoid formation was observed under brightfield conditions before and after NaI treatment. Images were collected at the beginning (t = 0) and end (t = 24 h) of the treatment, using the same imaging parameters. Organoid size was assessed by measuring the area of each organoid in ImageJ. For ER stress induction, thyroid organoids were exposed to a culture medium containing 10 µg/mL tunicamycin (Tm) for 24 h.

### 2.3. Immunohistochemical and Whole-Mount Immunofluorescence Staining

Fresh thyroid organoids or tissues were fixed, and 4 µm paraffin sections were stained with H&E according to standard protocols. For immunohistochemistry, after deparaffinization, antigen retrieval, and blocking with 5% goat serum in PBS, sections were incubated with primary antibody (1:200) for TSH-R (Abcam, Cambridge, UK, ab202960) or RFP (Rockland, Gilbertsville, PA, USA, 600-401-379), followed by incubation with anti-Rabbit secondary antibody (1:100, Beyotime, Xi’an, China, P0615-1).

For whole-mount immunofluorescence staining, thyroid organoids were fixed with 4% PFA at 4 °C for 1 h, followed by triple PBS washing. After blocking and permeabilization, organoids were then incubated with anti-sodium iodide symporter (anti-NIS) primary antibody (1:100, Proteintech, 24324-1-AP) overnight at 4 °C. After that, organoids were treated with anti-Rabbit secondary antibody (1:100, Beyotime, P0615-1), followed by incubation with DAPI (1:1000, Beyotime, C1002). Confocal microscopy image acquisition and analysis were performed as described before [20].

### 2.4. Measurement of mRNA and Protein Expression

TRIzol (Invitrogen, San Diego, CA, USA, 15596018) was used to isolate RNA. PrimeScript^TM^ RT reagent Kit (Takara, Otsu, Japan, RR037A), Hieff^®^ qPCR SYBR^®^ Green Master Mix (YEASEN, Shanghai, China, 11203ES08), and QuantStudio 6 (ThermoFisher, Carlsbad, CA, USA) were used for RT-PCR. The primers used in RT-PCR are listed in Table 2. The cycle threshold (Ct) value was calculated, and β-actin was used for normalization. For Western blot analysis, anti-XBP1 (1:1000, Proteintech, 24168-1-AP), anti-XBP1s (1:1000, CST, 40435S), anti-HSP90 (1:1000, CST, 4874S), anti-p-IRE1α (1:1000, NB100-2323, Novus Biologicals, Centennial, CO, USA), anti-IRE1α (1:1000, 3294, Cell Signaling Technology, Danvers, MA, USA), anti-XBP1s (1:1000, 40435, Cell Signaling Technology), anti-BiP (1:20,000, 66574-1-lg, proteintech), anti-p-PERK (1:1000, AP0886, Abclonal, Wuhan, China), anti-PERK (1:1000, 3192, Cell Signaling Technology), anti-ATF4 (1:2000, 60035-1-lg, proteintech), and anti-CHOP (1:1000, 2895, Cell Signaling Technology) were applied.

### 2.5. Transfection

The full-length human XBP1s expression plasmid was a gift from Prof. Yong Liu (Wuhan University). ShRNA against human XBP1, designed by RNAi software (broadinstitute.org, accessed on 1 August 2021) (Table 3), was used for cloning into the pLKO.1 vector at Age I and EcoR I sites. Then pECMV-hXBP1s or pLKO.1-sh-hXBP1 were transfected by using Lipofectamine 3000 (Invitrogen, L3000015).

### 2.6. ChIP-PCR Assay

An EZ Magna ChIP G Kit (Millipore, Billerica, MA, USA, 17-409) was used for chromatin immunoprecipitation (ChIP) assay. Cells were treated by 10 µg/mL tunicamycin for 6 h, and immunoprecipitation was applied by using 2 µg anti-mouse IgG (Millipore, 12-371B) or anti-XBP1s antibody (1:1000, CST, 40435S) or anti-H3K9me3 antibody (Millipore, 05-1242) or anti-H3K27me3 antibody (Abcam, ab6002). Agarose gel electrophoresis was performed for PCR products. Primer sequences for ChIP-PCR are provided in Table 4.

### 2.7. Statistical Analysis

For statistical analysis, GraphPad Prism 8 (GraphPad, La Jolla, CA, USA) was applied. A value of *p* < 0.05 was considered to be significant.

## 3. Results

### 3.1. Excessive Iodide Intake Downregulates Genes Involved in TH Synthesis in Mouse Thyroid

**Figure 1:**
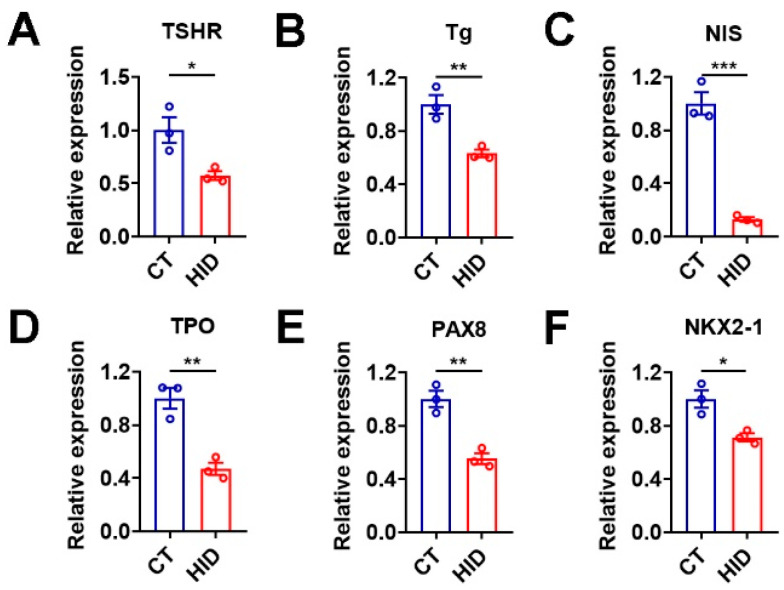
Excessive iodide intake downregulates the mRNA expression of genes involved in thyroid hormone (TH) synthesis in mouse thyroid. (**A**–**F**) Relative mRNA levels of genes involved in TH synthesis, including TSHR (**A**), Tg (**B**), NIS (**C**), TPO (**D**), PAX8 (**E**), and NKX2-1 (**F**), in the thyroid of C57 mice after exposure to excess iodide. Individual data points represent pooled samples, each containing thyroids pooled from three mice in each group. HID, high-iodide diet group; CT, control group. Data are presented as mean ± SEM. Statistical significance was determined by Student’s *t*-test; * *p* < 0.05, ** *p* < 0.01, and *** *p* < 0.001.

To explore the effect of iodide excess on thyroid physiology, mice were exposed to sodium iodide in their drinking water for 3 months. The mRNA expression of genes related to TH synthesis, including thyrotropin receptor (TSHR), thyroglobulin (Tg), sodium/iodide symporter (NIS), thyroid peroxidase (TPO), paired box gene 8 (PAX8), and NK2 homeobox 1 (NKX2-1, also known as TTF1), was significantly reduced in the thyroid of mice after excessive iodide treatment (Figure 1A–F). These results indicate that excessive iodide intake suppresses the mRNA expression of these genes related to TH synthesis in mouse thyroid.

### 3.2. Sodium Iodide Has No Effect on Genes Involved in TH Synthesis in Cultured Cell Lines

To further explore the effect of iodide excess on genes related to TH synthesis, we treated non-tumorigenic human thyroid follicular epithelial Nthy-ori 3-1 cells [21] and human papillary thyroid-cancer-derived BCPAP cells [22] with different concentrations of sodium iodide. Sodium iodide treatment did not affect the mRNA levels of TSHR, Tg, NIS, and TPO in these two cell lines at any of the concentrations tested (Supplementary Figure S1A,B). Since monolayer culture systems lack the natural follicle structure, these cell lines may be inappropriate for investigating the iodide effect. Thus, an alternative culture system is urgently needed.

### 3.3. Three-Dimensional Culture Enables Thyrocytes to Form Organoids with Follicle-like Structures

**Figure 2:**
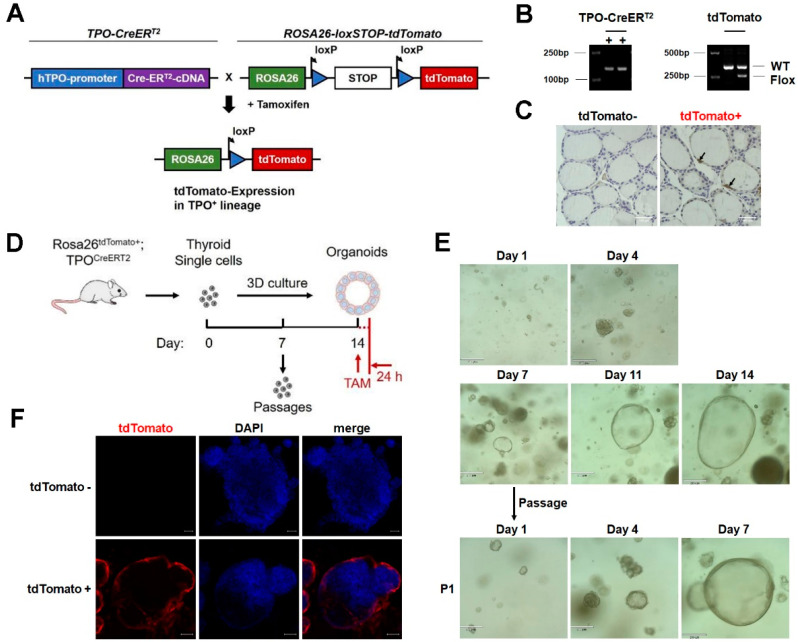
Three-dimensional (3D) culture enables the thyrocytes to form organoids with follicle-like structures. (**A**,**B**) Schematic diagram for the generation of Rosa26^tdTomato/+^;TPO^CreERT2/+^ mice (**A**) and genotyping of Rosa26^tdTomato/+^;TPO^CreERT2/+^ mice (**B**). (**C**) Representative images of tdTomato immunohistochemical staining of thyroid tissues from Rosa26^tdTomato/−^;TPO^CreERT2/+^ mice and Rosa26^tdTomato/+^;TPO^CreERT2/+^ mice treated with tamoxifen. Scale bars: 100 µm. (**D**) Schematic diagram of thyroid organoids culture and passaging. TAM, tamoxifen. (**E**) Light microscopy pictures of thyroid organoids developing from a single cell over the course of 14 days (upper panel) and follicles-like structure formation capacity upon passaging in 3D culture (*n* = 9). Scale bars: 210 µm. (**F**) TdTomato immunofluorescent staining of thyroid organoids from Rosa26^tdTomato/−^;TPO^CreERT2/+^ (upper panel) and Rosa26^tdTomato/+^;TPO^CreERT2/+^ (lower panel) mice. tdTomato (Red); nuclear staining with DAPI (Blue). Scale bars: 50 µm.

As cultured organoids normally display the structures and retain functional hallmarks of real organs [23], we attempted to generate thyroid organoids as a model system to explore the effect of iodide on thyroid physiology. To facilitate the evaluation of organoid development, we employed inducible TPO-CreERT2 mice and ROSA26-tdTomato mice to generate Rosa26^tdTomato/+^;TPO^CreERT2/+^ mice, in which the tdTomato fluorescence could be induced in TPO-expressing thyrocytes upon tamoxifen treatment (Figure 2A–C). The dissociated primary cells from the thyroid of Rosa26^tdTomato/+^;TPO^CreERT2/+^ mice were seeded directly in Matrigel for primary three-dimensional (3D) culture (Figure 2D). These cells could spontaneously grow and form thyroid-follicle-like structures in 3D culture (Figure 2E, upper panels). Moreover, the thyroid-follicle-like structures could also be obtained by using in vitro passaged primary cells (Figure 2D,E, lower panels), suggesting that the 3D-cultured primary cells retained the stem cell population. Furthermore, tdTomato^+^ cells could be observed in follicle-like structures after tamoxifen treatment, indicating that these organoids were bona fide thyrocytes expressing TPO (Figure 2F).

### 3.4. Three-Dimensional Cultured Murine Thyroid Organoids Retain the Thyroid Function

**Figure 3:**
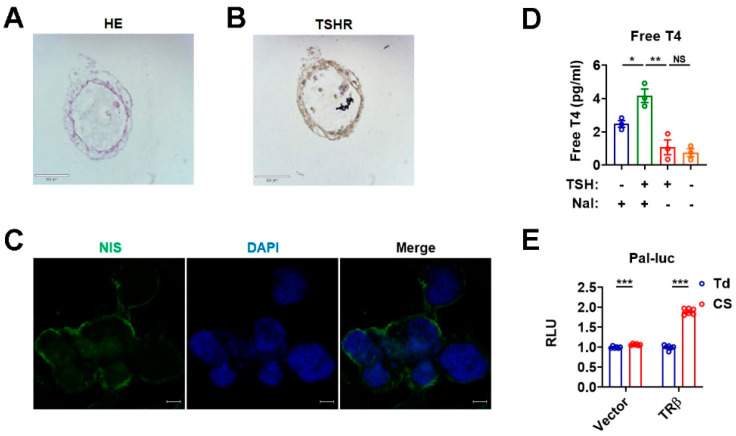
Three-dimensional cultured murine thyroid organoids retain the thyroid function. (**A**,**B**) Representative images of hematoxylin and eosin (H&E) staining (**A**) and TSHR immunohistochemical staining (**B**) of thyroid organoids. TSHR, thyrotropin receptor. Scale bars: 50 µm. (**C**) Immunofluorescent staining of NIS in thyroid organoids. NIS (green), nuclear staining with DAPI (blue). Scale bars: 50 µm. (**D**) The levels of free T4 in the culture supernatants of murine thyroid organoids exposed to NaI (100 nM) with or without hTSH (1 mU/mL) (*n* = 3). (**E**) Dual luciferase assay in 293T cells co-transfected with vector or TRβ and Pal-luc plasmids, maintained in TH-deficient (Td) medium, or treated with culture supernatants (CS) from the thyroid organoids (*n* = 6). Data are presented as mean ± SEM. Statistical significance was determined by Student’s *t*-test; * *p* < 0.05, ** *p* < 0.01, and *** *p* < 0.001. NS, not significant.

To test whether these tdTomato^+^ cells with follicle-like structures and certain polarity were functional, we analyzed their capability of hormone production in response to hTSH and sodium iodide treatment. In line with a positive staining of TSHR and NIS, respectively, for these cells with thyroid follicle-like structures (Figure 3A–C), hTSH treatment increased the free T4 level in the culture medium in the presence of sodium iodide (Figure 3D). Consistently, the culture medium of the organoids treated with both hTSH and sodium iodide could increase the reporter activity of Pal-luc in the presence of TRβ (Figure 3E), further confirming that these cells with thyroid-follicle-like structures were composed of functional thyrocytes in terms of hormone production. Collectively, our results suggest that the thyroid organoids obtained by 3D culture can display architectures and functionalities similar to in vivo thyroid gland and can serve as a suitable model to investigate the iodide effects in vitro.

### 3.5. Effects of Iodide Excess on Genes Involved in TH Synthesis Can Be Mimicked in Murine Thyroid Organoids

**Figure 4:**
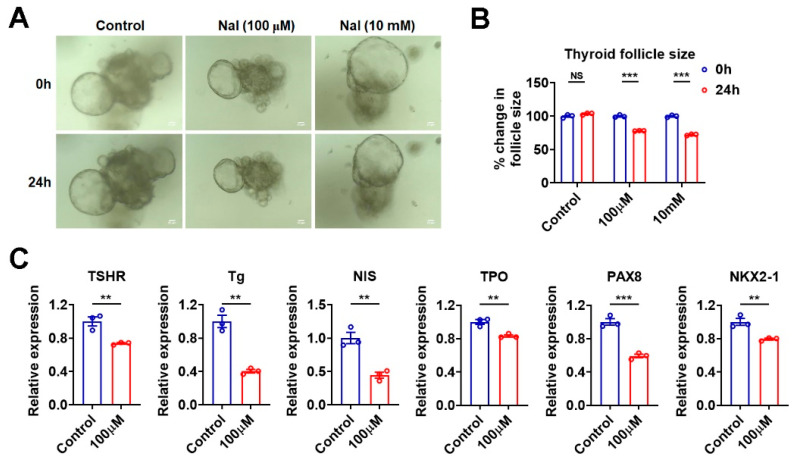
Effects of iodide excess on genes involved in TH synthesis can be mimicked in murine thyroid organoids. (**A**) Light microscopy pictures of thyroid organoids treated with PBS (as control) or excessive iodide for 24 h (*n* = 3). Scale bars: 50 µm. (**B**) The percent change in follicle size was quantified from thyroid organoids, as in (**A**). (**C**) Relative mRNA levels of TSHR, Tg, NIS, TPO, PAX8, and NKX2-1 in thyroid organoids treated with PBS (as control) or 100 µM NaI for 24 h (*n* = 3). Data are presented as mean ± SEM. Statistical significance was determined by Student’s *t*-test; ** *p* < 0.01, and *** *p* < 0.001. NS, not significant.

To test whether the effects of iodide excess on mouse thyroid could be observed in the murine thyroid organoids, we treated the cultured thyroid organoids with high concentrations of sodium iodide. In accordance with an in vitro study of porcine thyroid glands [24], we found that cultured thyroid follicles shrank upon excessive sodium iodide treatment regardless of the concentration used (Figure 4A,B). Moreover, the mRNA levels of TSHR, Tg, NIS, TPO, PAX8, and NKX2-1 were all significantly decreased upon sodium iodide treatment at 100 μM (Figure 4C), which mimicked the effect of excessive iodide intake on mouse thyroid (Figure 1). These results indicate that the generated thyroid organoids can serve as a proper in vitro culture model for the study of iodide excess.

### 3.6. Iodide Excess Activates XBP1 in Murine Thyroid and Thyroid Organoids

**Figure 5:**
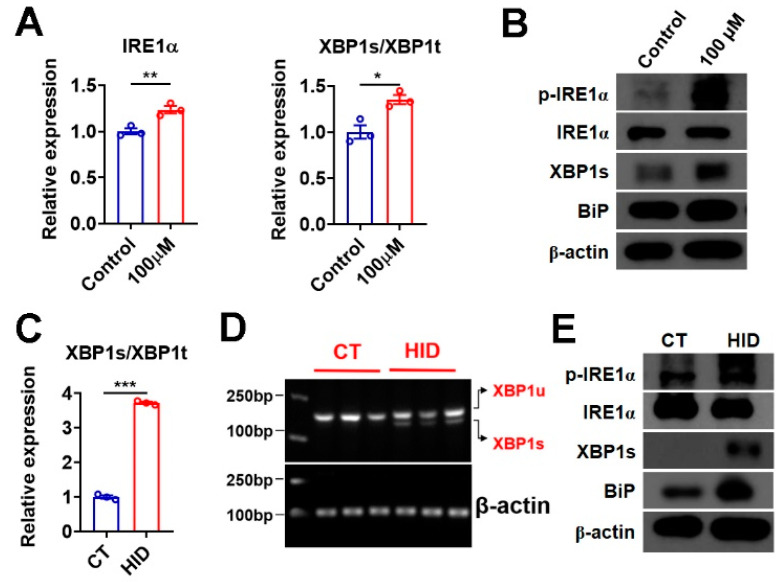
Iodide excess activates XBP1 in murine thyroid and thyroid organoids. (**A**) Relative mRNA levels of IRE1α (left panel) and the ratio of XBP1s (the spliced active form of XBP1) to XBP1t (total XBP1) (right panel) in murine thyroid organoids incubated with PBS (as Control) or 100 µM NaI for 24 h (*n* = 3). (**B**) Protein levels of p-IRE1α, IRE1α, XBP1s, and BiP in murine thyroid organoids incubated with PBS (as control) or 100 µM NaI for 24 h. Three samples pooled in each group. (**C**,**D**) The ratio of XBP1s (the spliced active form of XBP1) to XBP1t (total XBP1) (**C**) and agarose gel electrophoresis of XBP1 splicing (**D**) in thyroid tissues of C57 mice after exposure to excessive iodide. Individual data points represent pooled samples, each containing thyroids pooled from three mice in each group (**C**,**D**). (**E**) Protein levels of p-IRE1α, IRE1α, XBP1s, and BiP in thyroid tissues of mice after exposure to excessive iodide. Three samples pooled in each group. HID, high-iodide-diet group; CT, control group; XBP1u, unspliced XBP1. Data are presented as mean ± SEM. Statistical significance was determined by Student’s *t*-test; * *p* < 0.05, ** *p* < 0.01, and *** *p* < 0.001.

As thyroid follicles are believed to be sensitive to factors that can trigger ER stress, we then tested whether iodide excess would affect ER stress in murine thyroid organoids. The mRNA expression of IRE1α and the ratio of spliced form to total XBP1 (XBP1s/XBP1t) were both increased in thyroid organoids after sodium iodide treatment (Figure 5A). Accordingly, the phosphorylation of IRE1α, the XBP1s protein levels, and the expression of BiP, an XBP1 target gene, were increased in these thyroid organoids after the treatment of sodium iodide (Figure 5B and Supplementary Figure S2A). We did not observe any alterations in the mRNA levels of key genes in the other two UPR branches initiated by PERK (ATF4 and its target gene CHOP) and ATF6 (ATF6 and its target gene Herpud1), the phosphorylation of PERK, the ATF4 and CHOP protein levels in these organoids after treatment (Supplementary Figure S2A,B). Consistently, increased splicing of XBP1 was observed in the thyroid of mice with excessive iodide intake (Figure 5C). The semi-quantitative RT-PCR analysis of XBP1s and unspliced XBP1 (XBP1u), using a single pair of primers, further confirmed that sodium iodide treatment could induce the splicing of XBP1 (Figure 5D). Increased phosphorylation of IRE1α, XBP1s protein levels, and BiP expression could be detected in these mice with excessive iodide intake (Figure 5E). Similar to what we observed in cultured thyroid organoids, we could not observe any changes in the mRNA expression of ATF4, CHOP, ATF6, and Herpud1; the phosphorylation of PERK, the protein levels of ATF4 and CHOP in the thyroid of mice after treatment (Supplementary Figure S2C,D). Based on these data, we speculate that iodide excess mainly activates the IRE1α-XBP1-mediated UPR branch in the thyroid gland.

### 3.7. Activation of XBP1 Mimics the Iodide Excess Effect on Genes Related to TH Synthesis in Murine Thyroid Organoids

**Figure 6:**
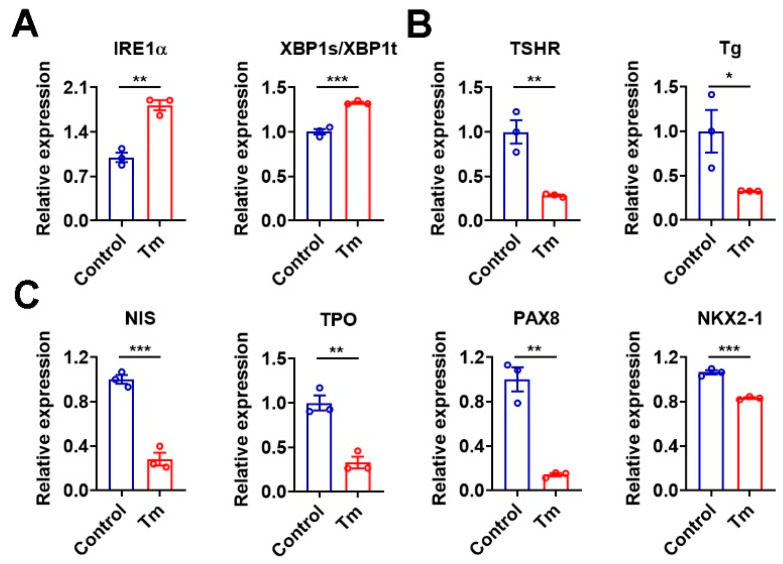
Activation of XBP-1 mimics the iodide excess effect on genes involved in TH synthesis in murine thyroid organoids. (**A**) Relative mRNA levels of IRE1α (left panel) and ratio of XBP1s to XBP1t (right panel) in murine thyroid organoids treated with DMSO (as control) or Tm for 24 h (*n* = 3). (**B**,**C**) Relative mRNA levels of TSHR, Tg (**B**), NIS, TPO, PAX8, and NKX2-1 (**C**) in thyroid organoids treated with DMSO (as control) or Tm for 24 h (*n* = 3). Tm, tunicamycin. Data are presented as mean ± SEM. Statistical significance was determined by Student’s *t*-test; * *p* < 0.05, ** *p* < 0.01, and *** *p* < 0.001.

To test whether the dysregulation of genes involved in TH synthesis under the iodide-excess condition was caused by ER stress via the activation of XBP1, we activated the XBP1 by using Tm, an ER stress inducer. As expected, Tm treatment resulted in increased IRE1α mRNA and an increased XBP1s/XBP1t ratio in murine thyroid organoids (Figure 6A). Meanwhile, the mRNA levels of TSHR, Tg, NIS, TPO, PAX8, and NKX2-1 were all reduced in these thyroid organoids after Tm treatment (Figure 6B,C), which phenocopied the effect of sodium iodide administration. The regulation of these genes by XBP1 was further confirmed in Nthy-ori 3-1 cells overexpressing the active form of XBP1 (Supplementary Figure S3A–C). In line with these data, the knockdown of XBP1 by specific shRNA could upregulate the mRNA levels of these genes (Supplementary Figure S4A–C). Based on these data, we propose that iodide excess suppresses the expression of genes related to TH synthesis pathway, which involves XBP1-mediated action.

### 3.8. XBP1 Transcriptionally Represses the Genes Involved in the Synthesis of TH

**Figure 7:**
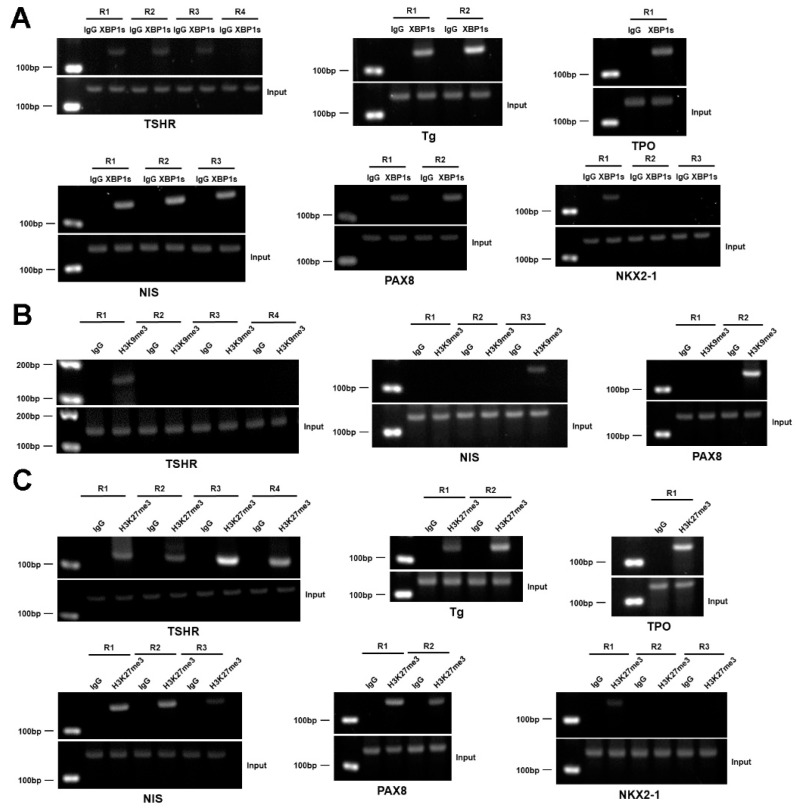
ChIP analysis of XBP1 recruitment and H3K9me3/H3K27me3 levels on the promoters of genes involved in TH synthesis. (**A**–**C**) ChIP-PCR analysis of the recruitment of XBP1s (**A**) and H3K9me3 (**B**) and H3K27me3 (**C**) on the promoters of TSHR, Tg, TPO, NIS, PAX8, and NKX2-1 in Nthy-ori 3-1 cells after tunicamycin treatment for 6 h.

To test whether XBP1s regulates the expression of these genes related to TH synthesis as a transcription factor, we performed ChIP assay in Nthy-ori 3-1 thyrocytes and observed the recruitment of XBP1s to the promoter regions of TSHR, Tg, TPO, NIS, PAX8, and NKX2-1, which contain putative XBP1s binding sites (Figure 7A and Supplementary Figures S5 and S6A). As H3K9me3 and H3K27me3 are normally correlated with transcriptional repression [25], we then tested whether there is an occupancy of H3K9me3 or H3K27me3 on these promoters. In agreement with the notion that XBP1s negatively regulates the mRNA expression of these genes, ChIP analysis revealed an occupancy of H3K9me3 and H3K27me3 on the promoters of TSHR, NIS, and PAX8, while an occupancy of H3K27me3 but not H3K9me3 was observed on the promoters of Tg, TPO, and NKX2-1 (Figure 7B,C and Supplementary Figure S6B,C). These data suggest that TSHR, Tg, TPO, NIS, PAX8, and NKX2-1 are direct target genes of XBP1s, and XBP1s represses the transcription of these genes via a mechanism involving H3K27me3 and/or H3K9me3. Taken together, we speculate that iodide excess inhibits the TH synthesis pathway, which involves XBP1-mediated action (Supplementary Figure S7).

## 4. Discussion

Iodine is a micronutrient which is needed for TH synthesis. The recommended iodine intake for normal adults is 150 μg/day. Although iodine excess can potentially cause thyroid dysfunction in susceptible subjects, it is widely accepted that iodine excess is usually well-tolerated. In general, 1100 μg/day is considered a tolerable upper limit at which adverse effects can be ignored for normal adults [3]. However, iodine content is thousands of times higher than this amount in iodine-rich food, supplements, and medications, as well as iodinated contrast agents. Occasionally, iodine-excess-induced unwanted effects can be observed after a single exposure to iodine-rich materials [3]. Thus, understanding the role of iodine in thyroid physiology and iodine excess in thyroid disorder will provide not only better recommendations for susceptible subjects but also a basis for the guidelines for the clinical management of diseases or the usage of iodine-containing contrast agents under certain circumstances.

A high amount of iodide-induced transient suppression of TH synthesis is characteristic of the acute Wolff–Chaikoff effect. Inhibition of NIS expression can serve as a mechanism to escape from or adapt to this effect, thus reducing the intracellular iodide content and resuming the near-normal TH synthesis [6]. In addition to NIS, iodide excess can also reduce the mRNA of other genes related to the synthesis of TH, such as TSHR, TPO, and Tg in thyrocytes [26]. Although transcriptional and post-transcriptional mechanisms have been proposed for the Wolff–Chaikoff effect [5,6,27], the underlying mechanisms are not fully understood. A recent report showed that ER stress could inhibit genes for TH synthesis in cultured rat thyrocytes [15]. Here, we found that, in response to iodide excess, the downregulation of TSHR, Tg, NIS, and TPO was accompanied by IRE1α-XBP1 rather than PERK or ATF6-mediated UPR branch activation. Moreover, TSHR, Tg, TPO, NIS, PAX8, and NKX2-1 are all direct target genes of XBP1. Thus, our study suggests that XBP1-mediated regulation is critically involved in the thyroidal adaptation to iodide excess through modulating TH synthesis pathways at a transcriptional level, although we cannot totally rule out the possibility that other mechanisms also can contribute to the effect of iodide excess.

Lacking a proper in vitro model system has hampered the research of thyroid physiology and pathology. In the current study, the action of sodium iodide on the genes involved in TH synthesis was only observed in cultured thyroid organoids but not in cultured cell lines (Supplementary Figure S1A,B), suggesting that follicle structure and functional polarity, which provide thyrocytes with normal cellular integration, are required for the regulation of TH synthesis by iodide. Importantly, by taking advantage of our mouse thyroid organoid culture system, we found that sodium iodide treatment could activate XBP1 by increasing its splicing, which phenocopied the effect of exposure to excess iodide in mice. Thus, we conclude that the murine thyroid organoid culture system is appropriate for the investigation of the iodide effect on thyroid function, including TH synthesis.

## Figures and Tables

**Table 1:** Primer information for genotyping.

Gene Name	Primer Sequence	Band Sizes
tdTomato P1	CTCTGCTGCCTCCTGGCTTCT	Wild type = 330 bp tdTomato = 250 bp
tdTomato P2	CGAGGCGGATCACAAGCAATA	
tdTomato P3	TCAATGGGCGGGGGTCGTT	
TPO-Cre F	CTAGCATCTTGACGGGCTATC	TPO = 130 bp
TPO-Cre R	AGGCAAATTTTGGTGTACGG	

**Table 2:** Primer information for RT-PCR.

Primer	Sequence	
mNIS	GCTCAGTCTCGCTCAAAACC	CGTGTGACAGGCCACATAAC
mTSHR	CTCTCTTACCCGAGCCACTG	TTGTCACCCGGATCTTCTTC
mTPO	TGACTTCCAGGAGCACACAG	GCAAGTTCAGTGATGCCAGA
mTg	TGTCCCACCAAGTGTGAAAA	CCAAGGAAAGCTTGTTCAGC
mPAX8	CAGAAGGCGTTTGTGACAATGA	TGCACTTTGGTCCGGATGAT
mNKX2-1	TCCAGCCTATCCCATCTGAACT	CAAGCGCATCTCACGTCTCA
mIRE1α	CGCACATGGCAGGATCAAG	TGCCCACTGCCAGCTTCT
mXBP1t	TGGCCGGGTCTGCTGAGTCCG	GTCCATGGGAAGATGTTCTGG
mXBP1s	CTGAGTCCGAATCAGGTGCAG	GTCCATGGGAAGATGTTCTGG
mXBP1 splicing	ACACGCTTGGGAATGGACAC	CCATGGGAAGATGTTCTGGG
mBip	ACTTGGGGACCACCTATTCCT	ATCGCCAATCAGACGCTCC
mATF4	CCTGAACAGCGAAGTGTTGG	TGGAGAACCCATGAGGTTTCAA
mCHOP	CTGGAAGCCTGGTATGAGGAT	CAGGGTCAAGAGTAGTGAAGGT
mATF6	ATTCTCAGCTGATGGCTGTCCAGT	TGGTAACTTCCAGGCGAAGCGTAA
mHerpud1	CCAAAGCAGGAAAAGCGACA	GGATTCAGCACCCTTTGTGC
mβ-actin	TACCACCATGTACCCAGGCA	CTCAGGAGGAGCAATGATCTTGAT
hNIS	CTCTGCGGGACTTTGCAG	ATCACCACGACCTGGAAC
hTSHR	AGCCACTGCTGTGCTTTTAAG	CCAAAACCAATGATCTCATCC
hTPO	GTCTGTCACGCTGGTTATGG	CAATCACTCCGCTTGTTGGC
hTg	CAGCAACCAGCTTTGGTCAC	AAGAGAACAGGTCGTGCTGG
hXBP1t	GACGGGACCCCTAAAGTTCTG	CTTCTTTCGATCTCTGGCAGTC
hXBP1s	CTGAGTCCGAATCAGGTGCAG	ATCCATGGGGAGATGTTCTGG
hXBP1 splicing	CCTGGTTGCTGAAGAGGAGG	CCATGGGGAGATGTTCTGGAG
hβ-actin	GATCATTGCTCCTCCTGAGC	ACTCCTGCTTGCTGATCCAC

**Table 3:** Primer information for shRNA of XBP1.

Primer	Sequence	
shScramble	CCGGTCCTAAGGTTAAGTCGCCCTCGC	AATTCAAAAACCTAAGGTTAAGTCGCCC
	GAGCGAGGGCGACTTAACCTTAGGTTT	TCGCTCGAGCGAGGGCGACTTAACCTTA
	TTG	GGA
shXBP1-1	CCGGAGATCGAAAGAAGGCTCGAATCT	AATTCAAAAAAGATCGAAAGAAGGCTCG
	CGAGATTCGAGCCTTCTTTCGATCTTTTT	AATCTCGAGATTCGAGCCTTCTTTCGAT
	TG	CT
shXBP1-2	CCGGGCGGTATTGACTCTTCAGATTCTC	AATTCAAAAAGCGGTATTGACTCTTCAG
	GAGAATCTGAAGAGTCAATACCGCTTTT	ATTCTCGAGAATCTGAAGAGTCAATACC
	TG	GC
shXBP1-3	CCGGGAACAGCAAGTGGTAGATTTACT	AATTCAAAAAGAACAGCAAGTGGTAGAT
	CGAGTAAATCTACCACTTGCTGTTCTTTT	TTACTCGAGTAAATCTACCACTTGCTGT
	TG	TC

**Table 4:** Primer information for ChIP-PCR.

Primer	Sequence	
TSHR R1	CTGCCATTCCCGATTCTTCC	CTGCACCCCTGGAGGATTCT
TSHR R2	CAGTCGACTCAACCACCGGA	AACTTCAGCTCCTCCTGGCC
TSHR R3	GAAGTTCTGCAGGACATTGG	CCCTCCTTTCCTCCTCTTTA
TSHR R4	TGGAGAGAACTAATGGGAGG	GCAACCTTGACCCAAACAAG
Tg R1	AAGGTGATAGAATTGCCCCA	TACTTTGGGTGAAGTGTGTG
Tg R2	CATGGTGAAGAGGAGGAGAA	AATGCAGGTTGGAGGCTAGT
NIS R1	AGAGAAGGGCCCTGAGATGA	ATGAACCCGAATAAGCGGAG
NIS R2	GCACTTATCATGTACCAGGC	GGGAGACATTCAGAGCTCAA
NIS R3	CTAGGTCTGGAGGCGGAGTC	TTCCGCTGTCCCATGCAGCC
TPO R1	TGAGGAGAGACGCAGGGAAT	AGTCAGTGTGTTCCAGTGCA
PAX8 R1	CTCACAATCTTACGTGTACC	TTAAGAAGGGGTCCACTGGT
PAX8 R2	CAAGGAGCAAGAAAGAGGGC	GAGCAGCACCTGAAGCCATT
NKX2-1 R1	AACCCTCTTCCTCCTAAGCA	TCTTGCTTCCCGGGAATAAC
NKX2-1 R2	AACAGACAGACGGGCACTCA	ACCCTCGCCCATCTCCCAGA
NKX2-1 R3	TTTGTTCGAGGTGGGGCAAC	TTTTTCAGTGGGCCACCCCC

## Data Availability

Data are available from the corresponding author upon reasonable request.

## Supplementary Materials

The following supporting information can be downloaded at: https://www.mdpi.com/article/10.3390/nu15040887/s1. Figure S1: Sodium iodide has no effect on the genes involved in thyroid hormone (TH) synthesis in cultured cell lines. Figure S2: Iodide excess does not activate PERK/ATF4 and ATF6 signaling pathways in murine thyroid organoids and murine thyroid. Figure S3: Overexpression of XBP1s downregulates the genes involved in TH synthesis. Figure S4: Knockdown of XBP1 upregulates the genes involved in TH synthesis. Figure S5: Putative XBP1 binding sites on the promoters of genes involved in TH synthesis. Figure S6: ChIP-qPCR analysis of XBP1 recruitment and H3K9me3/H3K27me3 levels on the promoters of genes involved in TH synthesis. Figure S7: Proposed working model.

## Abbreviations

Thyrotropin receptor (TSHR), sodium/iodide symporter (NIS), thyroid peroxidase (TPO) and thyroglobulin (Tg), and their transcriptional regulators, such as paired box 8 (PAX8) and NK2 homeobox 1 (NKX2-1, also known as TTF1).
