# Supplementary material for: Iodide Excess Inhibits Thyroid Hormone Synthesis Pathway Involving XBP1-Mediated Regulation
Source: Nutrients. 2023 Feb 9;15(4):887. doi: 10.3390/nu15040887 (PMC9967305; doi:10.3390/nu15040887)
Supplement: Supplementary file 1 [file nutrients-15-00887-s001.zip › nutrients-2131837-supplementary.pdf]

Supplementary Materials

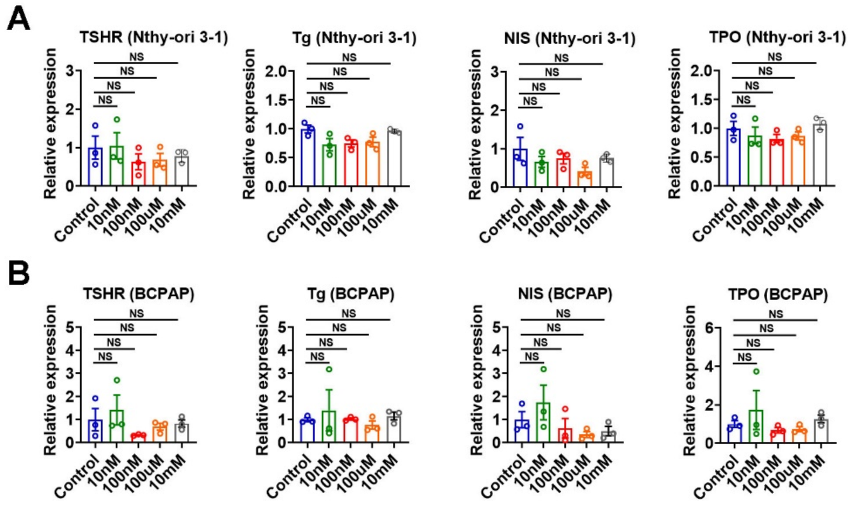

Figure S1. Sodium iodide has no effect on the genes involved in thyroid hormone (TH) synthesis in cultured cell lines. (A-B) Relative mRNA levels of genes involved in TH synthesis, including TSHR, Tg, NIS and TPO in the non-tumorigenic human thyroid follicular epithelial Nthy-ori 3-1 cells (A) and human papillary thyroid cancer-derived BCPAP cells (B) (n=3). Cells were incubated with indicated doses of NaI for 72 hours. Data are presented as mean ± SEM. Statistical significance is determined by Student's t-test. NS, not significant.

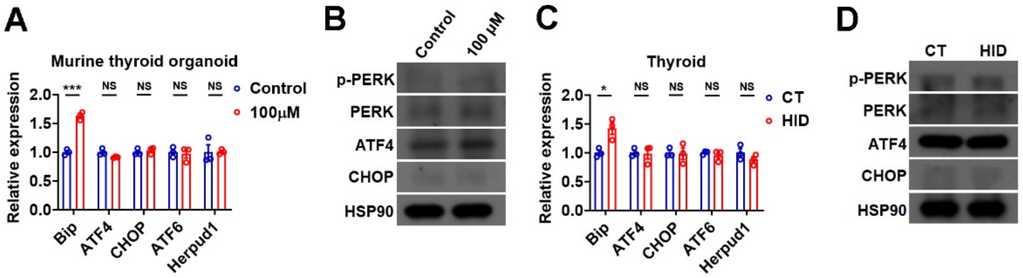

Figure S2. Iodide excess does not activate PERK/ATF4 and ATF6 signalling pathways in murine thyroid organoids and murine thyroid. (A) Relative mRNA levels of Bip, ATF4, CHOP, ATF6 and Herpud1 in murine thyroid organoids incubated with PBS (as Control) or 100 μM NaI for 24 hours (n=3). (B) Protein levels of p-PERK, PERK, ATF4 and CHOP in murine thyroid organoids incubated with PBS (as Control) or 100 μM NaI for 24 hours. Three samples pooled in each group. (C) Relative mRNA levels of Bip, ATF4, CHOP, ATF6 and Herpud1 in thyroid tissues of C57 mice after exposure to excessive iodide. Individual data points represent pooled samples, each containing thyroids pooled from three mice in each group. (D) Protein levels of p-PERK, PERK, ATF4 and CHOP in thyroid tissues of mice after exposure to excessive iodide. Three samples pooled in each group. HID: high-iodide diet group; CT: control group. Data are presented as mean ± SEM. Statistical significance is determined by Student's t-test. *$p<0.05$ and ***$p<0.001$. NS, not significant.

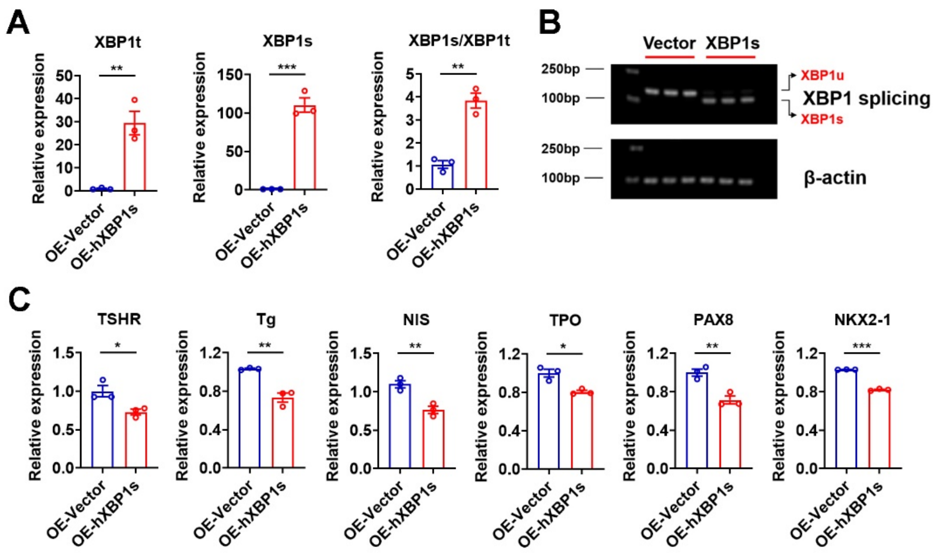

Figure S3. Overexpression of XBP1s downregulates the genes involved in TH synthesis. (A) Relative mRNA levels of XBP1t (total XBP1) (left panel) and XBP1s (the spliced active form of XBP1) (middle panel) and the ratio of XBP1s to XBP1t (right panel) in Nthy-ori 3-1 cells transfected with Vector or human XBP1s (hXBP1s) (n=3). (B) Agarose gel electrophoresis of XBP1 splicing in Nthy-ori 3-1 cells transfected with Vector or hXBP1s (n=3). XBP1u: unspliced XBP1. (C) Relative mRNA levels of TSHR, Tg, NIS, TPO, PAX8, and NKX2-1 in Nthy-ori 3-1 cells transfected with Vector or hXBP1s (n=3). Data are presented as mean ± SEM. Statistical significance is determined by Student's t-test. *$p<0.05$, **$p<0.01$ and ***$p<0.001$.

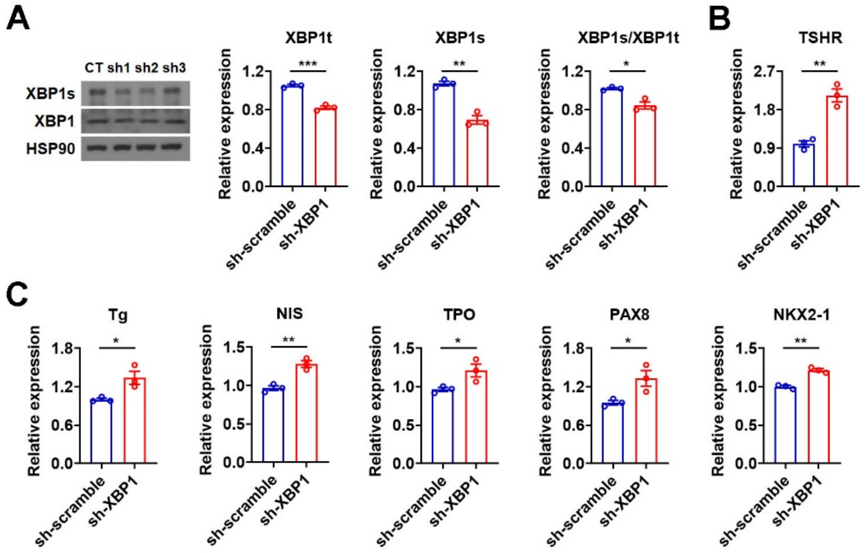

Figure S4. Knockdown of XBP1 upregulates the genes involved in TH synthesis. (A) XBP1s and XBP1 protein levels in Nthy-ori 3-1 cells transfected with three different sh-XBP1 (n=3), and relative mRNA levels of XBP1t (total XBP1) and XBP1s (the spliced active form of XBP1) and ratio of XBP1s to XBP1t in Nthy-ori 3-1 cells transfected with sh-XBP1 (n=3). (B-C) Relative mRNA levels of TSHR (B), Tg, NIS, TPO, PAX8, and NKX2-1 (C) in Nthy-ori 3-1 cells transfected with sh-XBP1 (n=3). Data are presented as mean ± SEM. Statistical significance is determined by Student's t-test. *p<0.05, **p<0.01 and ***p<0.001.

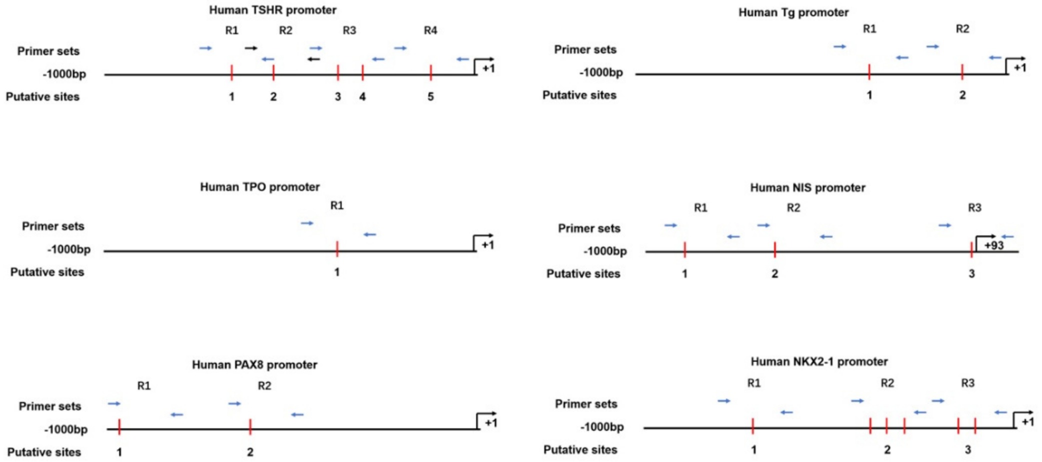

Figure S5. Putative XBP1 binding sites on the promoters of genes involved in TH synthesis. Arrows and numbers indicate PCR primer binding sites and putative XBP1 binding sites on the human promoters of TSHR, Tg, TPO, NIS, PAX8 and NKX2-1.

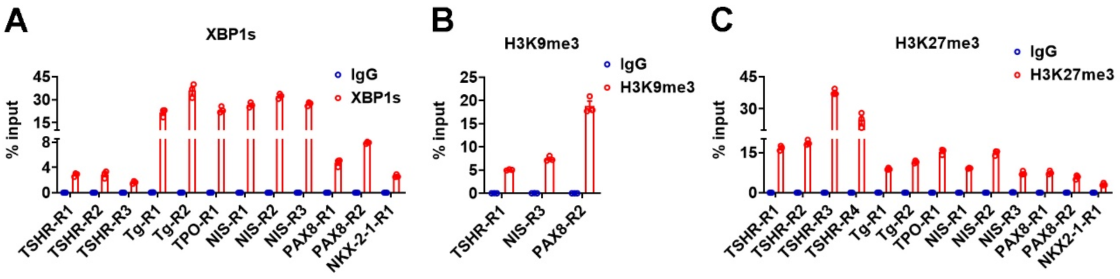

Figure S6. ChIP-qPCR analysis of XBP1 recruitment and H3K9me3/H3K27me3 levels on the promoters of genes involved in TH synthesis. (A-C) ChIP-qPCR analysis of the recruitment of XBP1s (A) and H3K9me3 (B) and H3K27me3 (C) on the promoters of TSHR, Tg, TPO, NIS, PAX8 and NKX2-1 in Nthy-ori 3-1 cells after tunicamycin treatment for 6 hours. Data are presented as mean ± SEM.

.

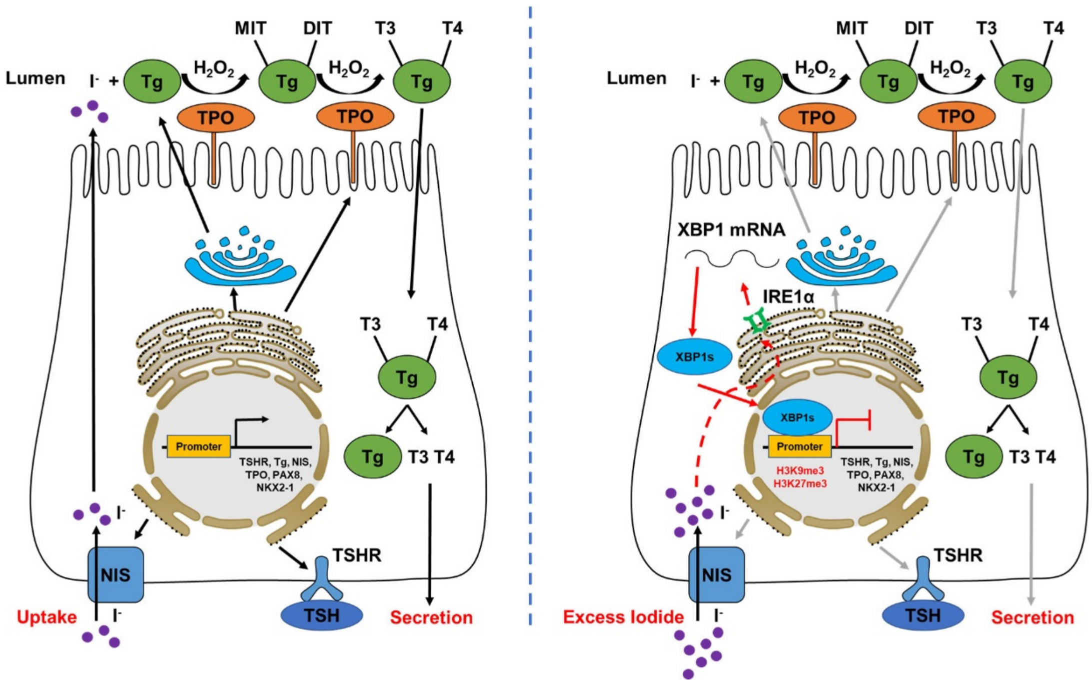

Figure S7. Proposed working model. The putative pathway involved in the transcriptional repression of TSHR, Tg, NIS, TPO, PAX8 and NKX2-1 by excessive iodide-activated IRE1α/XBP1s pathway. Under physiological conditions, the transcription of TSHR, Tg, NIS, TPO, PAX8 and NKX2-1 are positively regulated by TSH/TSHR signalling (left panel). Excess iodide uptake actives the IRE1α/XBP1s pathway, and XBP1s together with H3K9me3 ± H3K27me3 occupy the promoter regions of TSHR, Tg, NIS, TPO, PAX8 and NKX2-1, which represses the transcription of these genes (right panel).
